# UV-Light Exposure of Insulin: Pharmaceutical Implications upon Covalent Insulin Dityrosine Dimerization and Disulphide Bond Photolysis

**DOI:** 10.1371/journal.pone.0050733

**Published:** 2012-12-05

**Authors:** Manuel Correia, Maria Teresa Neves-Petersen, Per Bendix Jeppesen, Søren Gregersen, Steffen B. Petersen

**Affiliations:** 1 Department of Physics and Nanotechnology, Aalborg University, Aalborg, Denmark; 2 International Iberian Nanotechnology Laboratory (INL), Braga, Portugal; 3 NanoBiotechnology Group, Department of Biotechnology, Chemistry and Environmental Sciences, Aalborg University, Aalborg, Denmark; 4 Aarhus University Hospital, Aarhus Sygehus THG, Department of Medicine and Endocrinology MEA, Aarhus C, Denmark; 5 NanoBiotechnology Group, Department of Health Science and Technology, Aalborg University, Aalborg, Denmark; 6 The Institute for Lasers, Photonics and Biophotonics, University at Buffalo, The State University of New York, Buffalo, New York, United States of America; University of Hyderabad, India

## Abstract

In this work we report the effects of continuous UV-light (276 nm, ∼2.20 W.m^−2^) excitation of human insulin on its absorption and fluorescence properties, structure and functionality. Continuous UV-excitation of the peptide hormone in solution leads to the progressive formation of tyrosine photo-product dityrosine, formed upon tyrosine radical cross-linkage. Absorbance, fluorescence emission and excitation data confirm dityrosine formation, leading to covalent insulin dimerization. Furthermore, UV-excitation of insulin induces disulphide bridge breakage. Near- and far-UV-CD spectroscopy shows that UV-excitation of insulin induces secondary and tertiary structure losses. In native insulin, the A and B chains are held together by two disulphide bridges. Disruption of either of these bonds is likely to affect insulin’s structure. The UV-light induced structural changes impair its antibody binding capability and *in vitro* hormonal function. After 1.5 and 3.5 h of 276 nm excitation there is a 33.7% and 62.1% decrease in concentration of insulin recognized by guinea pig anti-insulin antibodies, respectively. Glucose uptake by human skeletal muscle cells decreases 61.7% when the cells are incubated with pre UV-illuminated insulin during 1.5 h. The observations presented in this work highlight the importance of protecting insulin and other drugs from UV-light exposure, which is of outmost relevance to the pharmaceutical industry. Several drug formulations containing insulin in hexameric, dimeric and monomeric forms can be exposed to natural and artificial UV-light during their production, packaging, storage or administration phases. We can estimate that direct long-term exposure of insulin to sunlight and common light sources for indoors lighting and UV-sterilization in industries can be sufficient to induce irreversible changes to human insulin structure. Routine fluorescence and absorption measurements in laboratory experiments may also induce changes in protein structure. Structural damage includes insulin dimerization via dityrosine cross-linking or disulphide bond disruption, which affects the hormone’s structure and bioactivity.

## Introduction

For several years, there has been significant interest on the effects of UV-light excitation on the structure and function of proteins [1]–[4]. This is particularly relevant for the food and pharmaceutical industries, and for the medical field where structural stability and activity of proteins as drugs or nutrients, is of nuclear importance. In the pharmaceutical industry, UV-light induced damage of proteins can occur during production, formulation, visual inspections, fill and finish operations, packaging, storage and delivery of the drug, since protein products will most likely be exposed to UV-light from natural or artificial light-sources [2], [5]. The same may occur during handling and administration of pharmaceuticals to patients, in hospitals and clinics (e.g. use of intravenous bags for administration of drugs) [2], [6]. A summary of possible UV-light induced reactions will be presented.

In proteins, the main targets of UV-light induced photo-degradation are the peptide backbone, tryptophan, tyrosine (Tyr, Y), phenylalanine, and cystine. In this work we will focus on Tyr photochemistry. The protein studied, insulin, does not contain any tryptophan residues. Furthermore, in this work insulin has been excited at 276 nm. At this wavelength and at neutral pH, Tyr absorption (ε_276 nm_ = 1362 cm^−1^.M^−1^ [7]) is higher than the absorption by cystine (ε_276 nm_ = 220 cm^−1^.M^−1^ for dimethylsulfide, model for cystine absorption [8]) or by phenylalanine (ε_276 nm_ = 3 cm^−1^.M^−1^ [7]).

Excitation of Tyr to higher electronic energy states is followed by distinct processes including relaxation by fluorescence to ground state, triplet state (^3^Tyr) formation, reaction with oxygen to form peroxy radicals, or excited state photochemical or photophysical processes, such as photoionization. Photoionization leads to the ejection of an electron from the residue, possibly yielding a solvated electron (e^−^_aq_), and a radical cation (^1^Tyr-OH**^.^** ^+^) followed by deprotonation resulting in formation of an uncharged radical (^1^Tyr-O**^.^**) [2], [3]. These processes are influenced by the pH of the solution, the temperature, the neighboring side-chains and the protein structure itself [2], [3]. Furthermore, in proteins Tyr can transfer their excited state energy to tryptophan [2]. A complete overview of the photophysical and photochemical mechanisms of Tyr can be found in our previous publication [2] and other literature [2], [9], [10].

**Figure 1:**
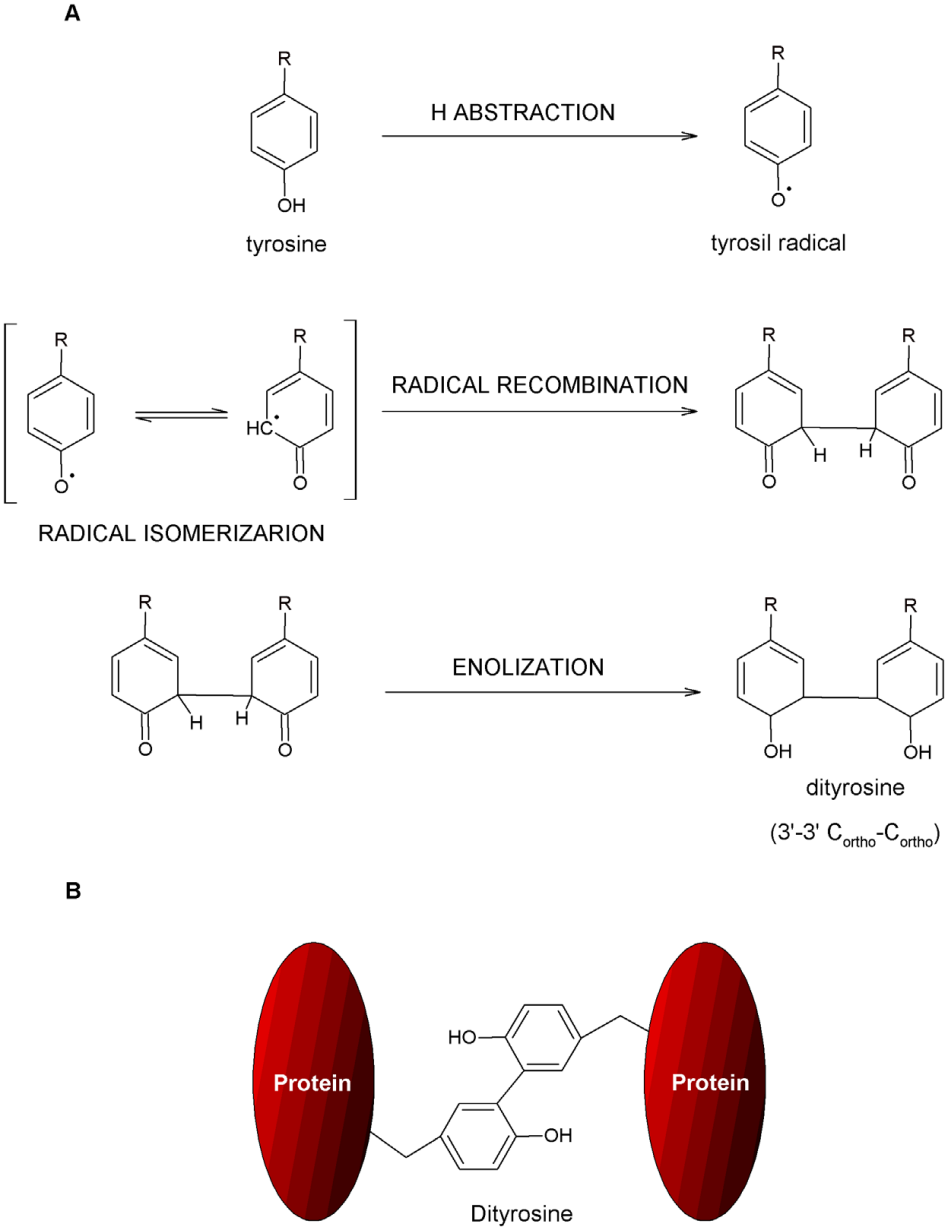
Molecular mechanisms of dityrosine cross-linking (C_ortho_-C_ortho_). ( A) Reaction mechanism for the formation of dityrosine. (B) Intermolecular dityrosine cross-linking in proteins.

The tyrosine radical ^1^Tyr-O**^.^** can also be involved in cross-linking through the ortho position leading to the formation of dityrosine (see Figure 1A) [2], [11]. Dityrosine is formed upon radical isomerization followed by diradical reaction, and finally enolization [12], [13]. Dityrosine is found in numerous proteins as a result of aging [13], exposure to oxygen free radicals [13], [14], nitrogen dioxide, peroxynitrite, and lipid hydroperoxides [13], enzymatic reaction with peroxidases [13], [15], [16], γ-irradiation [17], and UV-irradiation [18]–[20]. In these cases dityrosine cross-linking (C_ortho_-C_ortho_) can be either intramolecular or intermolecular [2], [12], [20] (see Figure 1B). Dityrosine is one of the specific markers for protein oxidation. The intermolecular cross-linking may result in protein aggregation [14], [15], [21] (See Figure 1B). Dityrosine formation upon UV-irradiation has been shown with fluorescence spectroscopy in L-dityrosine solutions [22], calmodulin [18], [20] and elastin hydrolysates (1 h excitation at 254 nm, power fluency of 43.8 W.m^−2^) [19]. The yield of dityrosine formation obtained in UV-irradiation of calmodulin (which contains a perfectly oriented Tyr pair and no tryptophan nor cysteine (Cys, C) is low (intermolecular cross-linking). There is only ∼6% conversion of Tyr to dityrosine and the reaction appears to be self-limiting [20]. Dityrosine displays unique fluorescence properties emitting strongly at 400–409 nm [20]. The deprotonated and protonated forms of dityrosine have absorption maxima at ∼315 nm and ∼283 nm respectively [22], [23]. The pK_a_ of the phenolic hydroxyl groups ionized group that deprotonates first in free dityrosine is ∼7.0–7.1 [22], [23]. It is only the single deprotonated form of dityrosine (in which one of the two phenolic hydroxyl groups is deprotonated) that emits at 400–409 nm [22], [24]. When dityrosine in is in the singlet excited state it has an apparent pK_a_ below 3. Thus, dityrosine fluorescence can be also seen in solutions with pH below 7, upon excitation of the protonated form (max at ∼283 nm), because deprotonation of the first phenolic hydroxyl group takes place in the excited state [20].

In proteins, another important photochemical mechanism which follows Tyr excitation is the reduction of disulphide bridges (SS) [1]–[3]. The solvated electrons generated upon Tyr excitation (either through photoionization or biphotonic absorption in the triplet state) can be captured by cystines leading to the formation of RSSR**^.^** ^−^ (disulphide electron adduct) and likely SS breakage (schemes 1, 2) [3], [25]. Solvated electrons can also interact with the peptide chain creating hydroxide ions and ketyl radicals (scheme 3), which can propagate along the peptide chain [10], [26]. If a ketyl radical gets trapped by a disulfide bridge, this again results in a disulphide anion and likely SS breakage. ^3^Tyr can also transfer an electron to a nearby SS forming Tyr**^.^** ^+^ and RSSR**^.^** ^−^ (scheme 4) [11]. Protonation of the disulphide anion can also lead to SS disruption (scheme 5) [25]. Photolysis of SS can also take place through direct excitation of cystine residues at ∼254 nm, with similar formation of the electron adduct RSSR**^.^** ^−^

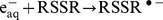
(1)

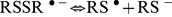
(2)

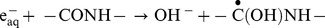
(3)

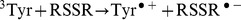
(4)

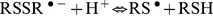
(5)


Reduction of SS upon UV excitation of aromatic residues has been shown for proteins such as cutinase and lysozyme [1], [3], [27], bovine serum albumin [28], [29], prostate specific antigen [30], alpha-lactalbumin [4] and antibody Fab fragments [31].

**Figure 2:**
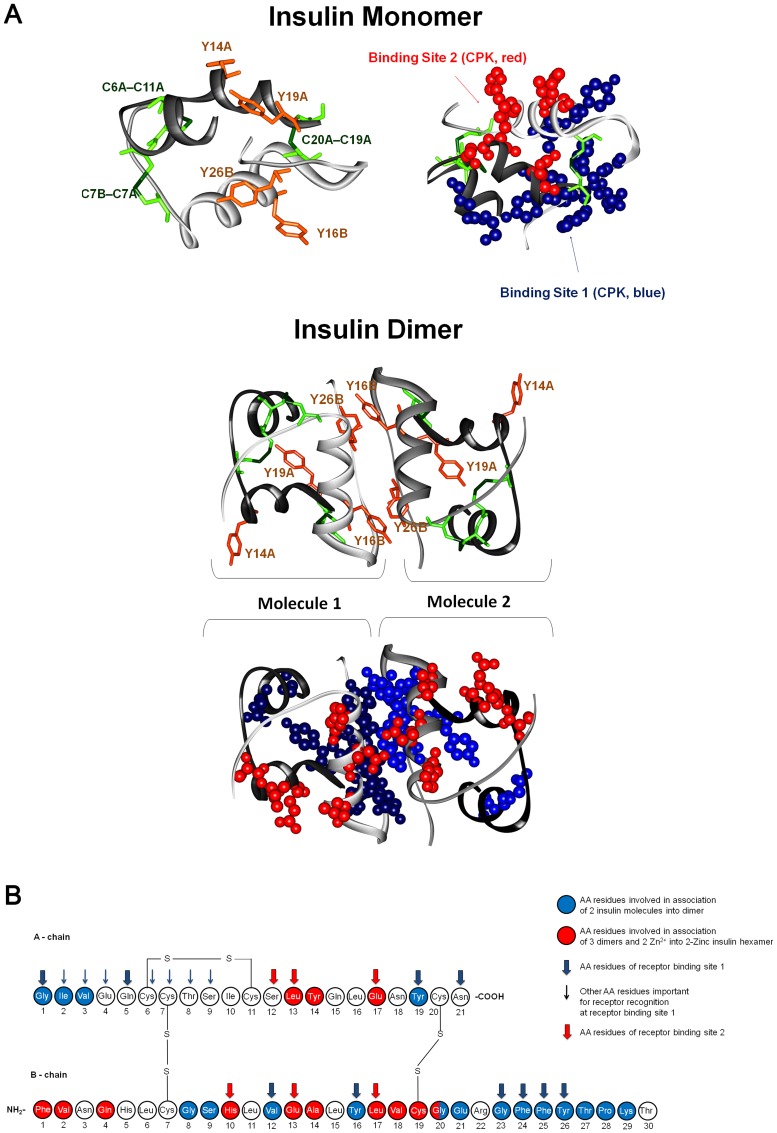
Tertiary and primary structures of insulin, dimer and 2Zn hexamer forms and receptor binding sites. (A) 3D structures of insulin monomer and dimer extracted from the crystallized structured of the 2Zn pig insulin hexamer (4INS.pdb) [37]. Insulin A chains of insulin are displayed in gray (two different shades in the insulin dimer) and B chains are displayed in black. The 4 Tyr residues of insulin (Y, orange) and the 6 Cys (C, green) involved in 3 disulphide bridges (SS) are displayed in orange and green, respectively (left-side structure for insulin monomer and top structure for insulin dimer). The two insulin binding sites are displayed in CPK (0.5 Å radius) in blue (binding site 1) and red (binding site 2) (right side structure for insulin monomer and bottom structure for insulin dimer). (B) Primary structure of human insulin. The amino acid residues involved in the association of 2 insulin molecules into a dimer (blue residues) and in the molecular assembly of 3 dimers into 2Zn insulin hexamer (red residues) are displayed. The amino acid residues belonging to insulin receptor binding sites 1 and 2 are indicated by wide blue and red arrows, respectively. Amino acid residues important for insulin receptor recognition of the binding site 1 but not belonging to this site are displayed with thin blue arrows (adapted from [36], information regarding dimer association, hexamer formation, and insulin receptor binding sites extracted from [35], [36], [38], [48]–[51]).

Insulin is a small and important peptide hormone for the mammals involved in processes such as cell growth, cell differentiation, membrane transfer of nutrients and metabolism [32]. Insulin interacts with the insulin receptors in muscle cells, liver, and adipose tissue [33]. It stimulates glucose uptake, glucose oxidation, glycolysis, glycogenesis, lypogenesis, protein synthesis and inhibits gluconeogenesis, lipolysis, fatty acid oxidation and protein degradation [32], [33]. In almost all the species, including human, it has 51 amino-acids and a molecular weight of ∼6000 Da [34]. The human insulin molecule is constituted by two polypeptide chains, an A chain and a B chain, containing 21 and 30 amino acid residues, respectively [35]. The two chains are linked together by two inter-chain SS (CysA7-CysB7 and CysA20-CysA19) and an additional SS connects CysA6 and CysA11 within the A chain (Figure 2A). The amino-acids of the two chains are also involved in numerous non-covalent interactions [35].

Although insulin binds to its receptor as a monomer, it assumes different forms both physiologically and in solution [36]–[38]. It forms dimers, double dimers (or tetramers), triple dimers (or hexamers), and higher-order aggregates [39]. The insulin form in solution dependents on e.g. insulin concentration, pH, and solvent composition, and metal ion concentration, which can also affect the charge and solubility of the insulin molecule [36], [39]. At physiologic pH, monomeric human insulin molecules in solution only exist at very low concentrations (<1×10^−9^ M), while dimers are formed at concentrations of about 1×10^−6^ M [36], [39]. In the blood, the physiological concentration of insulin is generally <1×10^−9^ M, which ensures the circulation of insulin and its biological effects as a monomer [36]. Insulin aggregation is initiated upon concentration increase and several models have been proposed for describing the phenomenon in the absence of metal ions [39], [40]. In the presence of zinc or other metal ions, three human insulin dimers readily associate into hexamers [39], [41]. 2Zn hexameric insulin assembles through the coordination of two zinc ions and has a similar structure to the natural storage species of the peptide in pancreatic beta-cells [41].

The stability of insulin molecule in either of its aggregation states has been extensively studied over the years, due to its importance for pharmaceutical preparations. Several studies have been reported on the effects of pH, temperature and other environmental stresses on the structure and function of the peptide (for review see: [42]) but little attention has been given to insulin exposure to UV-light. Here we show that prolonged exposure of human insulin to 276 nm UV-light has dramatic consequences on the absorption, fluorescence, structure and biofunctionality of human insulin. Continuous UV-exposure of insulin leads to SS breakage mediated by tyrosine excitation, covalent insulin dimerization via dityrosine cross-linking, and considerable losses in secondary and tertiary structural features. This structural modification impairs both insulin antibody recognition and biological function in *vitro*. For the first time to our knowledge we quantify the functional losses in insulin activity and we relate it to the irradiance levels used in our experiments. The effects of prolonged exposure of insulin pharmaceutical preparations to sunlight and artificial lighting,-and the implications for pharmaceutical industry are discussed.

## Materials and Methods

### Protein and Buffer Solutions

Human insulin was purchased from Sigma-Aldrich (product I2643, Sigma-Aldrich Danmark A/S, Copenhagen, Denmark) (MW = 5807.57 g.mol^−1^; Zinc, Zn ≤1%, dried basis). Stock solutions of insulin were prepared by dissolving the powder in 10 mM Phosphate Buffer pH 8.0 and stored at 4°C until use. Insulin concentrations were determined by Abs_276nm_ using a molar extinction coefficient of 1.0675 cm^−1^.(mg/ml)^−1^ [43]. Milli-Q water with conductivity below 0.2 µS.cm^−1^ was used.

### UV-excitation of Insulin Samples

Throughout the work insulin samples were excited on a RTC 2000 PTI spectrometer (Photon Technology International, Canada, Inc.347 Consortium Court London, Ontario, Canada) with a T-configuration, using a 75-W Xenon arc lamp coupled to a monochromator. For each experiment 2 mL of 17.7 µM insulin in 10 mM PBS pH 8.0 was placed in a quartz macro cuvette (1 cm path length) and continuously excited with UV-light over selected time periods. A fresh sample was used for each excitation session. Samples were magnetically stirred at 750 rpm in order to secure homogeneous excitation. Slits (bandpass) were always set to 5 nm. Lamp power at 276 nm was 142 µW and the same lamp was used throughout the experiments.

### UV-absorption Studies and Elmanńs Assay

Before analyzing the insulin samples by UV-absorption and quantifying the number of free thiol groups with the Elmann’s assay, insulin was continuously excited at 276 nm during 0.25 h, 0.5 h, 0.75 h, 1 h, 2 h, or 2.5 h. After each illumination, 1 mL of the illuminated solution was placed in 1 cm path length cuvette and an absorbance spectrum was recorded with UV/Visible spectrophotometer (UV1 VWR International–Thermo Electron Corporation, Thermo Fisher Scientific Inc. 81 Wyman Street Waltham, MA, USA). Absorbance spectra were acquired between 200 and 500 nm. A reference spectrum was acquired for a fresh insulin sample (time 0 h, control).

Detection of free thiol groups was carried out using the Ellman’s assay [1], [44]. Ellman’s reagent, 5,5′-dithiobis-2-nitrobenzoic acid (DTNB) was purchased from Molecular Probes (product D8451, Life Technologies, Naerum, Denmark). One hundred mM DTNB stock solution was prepared in DMSO and stored at 4°C. After each excitation session, 990 µL of illuminated insulin solution was mixed with an excess of DTNB (10 µL of 100 mM stock solution). The molar ratio DTNB/insulin was ∼57. Two minutes after mixing the two components, the absorbance intensity at 412 nm was monitored with a UV/Visible spectrophotometer (UV1 VWR International–Thermo Electron Corporation, Thermo Fisher Scientific Inc. 81 Wyman Street Waltham, MA, USA), using a 1 cm path length quartz cuvette. The sample was kept in the dark during the 2 min incubation period. Absorbance at 412 nm is due to the release of the product 2-nitro-5-thiobenzoate ion (TNB^2−^) and is proportional to the amount of thiol groups present in solution. Concentration of thiol groups was determined using an extinction molar coefficient for TNB^2−^ of 14150 M^−1^.cm^−1^ at 412 nm [44].

### Fluorescence Studies

Insulin samples were continuously excited at 276 nm during 0.5 h, 1 h, 1.5 h, 2.5 h, 3.5 h, and 7 h using the RTC 2000 PTI spectrometer setup as described above. Time-based fluorescent emission kinetic traces (emission fixed at 405 nm in detector 1 and 303 nm in detector 2) were obtained during continuous 276 nm excitation. Real-time correction was enabled in order to correct for oscillations in lamp intensity (gain set at 1 V).

Before and after each illumination, emission and excitation spectra were recorded. Emission spectra were acquired with 276 nm and 320 nm excitation. Excitation spectra were recorded with the emission fixed at 303 nm and 405 nm. The same emission and excitation spectra were acquired for the buffer. Slits (bandpass) were always set to 5 nm. During the whole procedure samples were magnetically stirred at 750 rpm in order to secure homogeneous excitation. Solution temperature was set at 21°C using a Peltier element at the cuvette holder location.

### Circular Dichroism Measurements

Circular Dichroism (CD) spectroscopy was used to monitor the relative changes in ellipticity after prolonged 276 nm excitation of insulin. Near- and far-UV CD spectra were immediately recorded after each excitation session described in the previous session (0 h, 0.5 h, 1 h, 1.5 h, 2.5 h, 3.5 h, and 7 h 276 nm excitation). Two hundred µl of illuminated insulin (17.7 µM in 10 mM PBS pH 8.0) was placed in a quartz microcuvette with a path length of 0.1 cm. Far-UV CD spectra (190–260 nm) were acquired using the following parameters: 1.0 nm band width, resolution 1.0 nm, 9 accumulations, scan speed 50 nm/min, 20 mdeg sensitivity, 4 s response time. Near-UV CD spectra (250–310 nm) were recorded using the following parameters: 10 nm band width, resolution 1.0 nm, 32 accumulations, scan speed 100 nm/min, 1 mdeg sensitivity, 4 s response time. Far-UV and near-UV CD were also acquired for a fresh insulin sample and for the buffer. The buffer signal was subtracted from all spectra. Each measurement was controlled by the JASCO J-700 hardware manager (JASCO Corporation, Ishikawa-cho Hachioji-shi, Tokyo, Japan). Temperature was kept constant at 21°C throughout all the measurements using a Peltier element.

### Radioimmunoassay

Insulin samples were continuously excited at 276 nm during 1.5 h or 3.5 h. As a control, in parallel, 2 mL of the same 17.7 µM insulin stock was placed in a quartz macro cuvette and left in the dark under 750 rpm during 1.5 or 3.5 h.

After excitation, the illuminated sample and the non-illuminated sample (positive control, PC) were diluted 500000 times. One hundred µL of each diluted sample was pre-incubated with a surplus (100 µL) of guinea pig anti-porcine insulin antibody serum (Novo Nordisk, Bagsvaerd, Denmark). During the incubation period (∼48 h) the guinea-pig anti porcine insulin antibodies bind to the intact insulin molecules forming a complex. After incubation an excess of labeled ^125^I-Insulin (100 µL with specific radioactivity of ∼371 mCi/mg, Novo Nordisk) is added to the reacting mixture and binds to the remaining antibodies in solution, forming a ^125^I-Insulin-antibody complex. To separate free insulin (non-bound) and ^125^I-Insulin excess from the antibody-bound insulin, the mixture is washed two times with 96% ethanol and subsequently FAM/ethanol. After mixing, antibody-bound insulin and antibody-bound ^125^I-Insulin precipitate immediately, while free insulin and free ^125^I-Insulin stay in the supernatant phase. The free ^125^I-Insulin concentration is then determined by measuring the radioactivity in the supernatant or the radioactivity of antibody-bound ^125^I-Insulin in the precipitate. The intensity of the radioactive-bound ^125^I-Insulin measured is directly proportional to the insulin concentration in the analyzed sample. Rat insulin is used as a standard sample. Insulin concentration is calculated from a standard curve obtained using the radioactivity intensity measurements of rat insulin (known concentrations) and their input in Multicalc.

### Glucose Uptake Assay

The effect of UV-light exposure of insulin on its *in vitro* function was tested using a bioassay based on glucose uptake in proliferating human skeletal muscle cells (SkMC) (Promocell, Heidelberg, Germany). The cells were prepared according to the Promocell manual and protocol. The cells were then seeded in 24 wells (0.3.10^6^ cells/well) containing 1 mL growth medium. Once 70–90% confluence was reached, the growth medium was replaced by 1 mL differentiation medium (Promocell) inducing the fusion of skeletal cells to myotubes with typical multinucleated syncytia. The differentiation medium was changed every 2^nd^ to 3^rd^ day. Once extensive formation of multinucleated syncytia was visible under the microscope (after 8–14 days), the cells were ready for the glucose uptake assay.

As a preliminary step for the glucose uptake assay, an insulin sample was continuously excited at 276 nm during 1.5 h using the RTC 2000 PTI spectrometer setup as described above. As a control, in parallel, 2 mL of the same 17.7 µM insulin stock was placed in a quartz macro cuvette and left in the dark under 750 rpm during 1.5 h. After excitation, the illuminated sample and the non-illuminated sample (positive control, PC) were diluted to the concentration to be used in the assay (∼100 nM).

After 8–14 days of culture in differentiation medium the 24 wells plate was washed twice with 1 mL Dullbecco’s Phosphate Buffer saline (D-PBS) without CaCl_2_ and MgCl_2_ (GIBCO, Paisley, UK). Afterwards 300 µL M-KRB supplemented with 0.1% BSA, 0.1 mM glucose (5 µCi/ml) or 1.5 µCi/well deoxy-d-glucose 2-[1,2–3H(M)], referred as deoxy-d-glucose (Nen, Perkin Elmer, 2740 Skovlunde, Denmark) was added to the plates. Additionally, for the wells where glucose uptake was being stimulated, insulin was supplemented to the medium. Three types of wells were prepared: one type with no insulin (control), a second with 100 nM illuminated insulin, and another with 100 nM of non-illuminated insulin (PC). In the last two cases insulin was directly diluted to the medium down to a concentration of 100 nM. Of the 24 wells, 6 wells were not supplemented with insulin (controls), 9 were stimulated with 100 nM of illuminated insulin, and 9 with the PC 100 nM insulin. The plates were kept on ice during the addition of medium. After 15 minutes incubation at 37°C the plates were placed on ice, and the incubation was stopped by washing twice with 1 ml M-KRB supplemented with 0.1% BSA and 50 mM glucose. Two hundred µl of 0.1 M NaOH was added to each well, and after 30 minutes at room temperature 100 µl was transferred to a 24 well counting plate (Wallac Oy, Turku, Finland) and 900 µL of Hisafe II scintillator (Perkin Elmer, 2740 Skovlunde, Denmark) was added. After ∼12 h the plates were counted using a micro beta counter TriLUX (Wallac Oy, Turku, Finland). Counts per minute is a direct measure of the glucose taken up by the muscle cells.

The assay was repeated maintaining all the experimental conditions except the following. Of the 24 wells, 6 wells were not supplemented with insulin (control), 12 were stimulated with 100 nM of illuminated insulin, and 6 wells with the PC 100 nM insulin.

### 3D Protein Structure

The crystallography data used for 3D protein structure display (Figure 2A), and distance calculations was extracted from the PDB file 4INS (2Zn hexameric pig insulin [37]). 3D structures of the insulin monomer and dimer were displayed using Accelrys Discovery Studio Visualizer 2.5. Distances between Tyr residues, and between Tyr residues and SS were calculated in Accelrys Discovery Studio Visualizer 2.5 using the “monitor” tool.

### Data Analysis

#### Absorbance Spectra

Absorbance spectra were first smoothed using a 7 points adjacent averaging and the baseline was corrected using the baseline tool of Origin Pro 8.0. Fitting of peaks at 276 and ∼314 nm was carried out using a 2 Gaussian function and the peak finder from Origin Pro 8.0. The peak at 276 nm was fixed. The fitting procedure was carried out between 260 and 400 nm.

#### Emission and Excitation Spectra

All spectra obtained were first Raman corrected by subtracting the corresponding spectra recorded for the buffer alone in solution. Emission spectra recorded upon 276 nm were smoothed using a 7 points adjacent averaging. Excitation spectra obtained with emission fixed at 405 nm were smoothed using a 5 points adjacent averaging.

Since the emission and excitation spectra recorded at excitation time 0 h were different for each excitation session (0.17 h, 0.33 h, 0.5 h, 0.75 h, and 1.5 h) a correction factor had to be applied to each spectrum. Each spectrum obtained after each excitation session (0.17 h, 0.33 h, 0.5 h, 0.75 h, and 1.5 h) was divided by the maximum intensity value of the corresponding 0 h emission or excitation spectrum. The emission spectra acquired upon 276 nm were divided respectively by the 300 nm emission intensity values of the corresponding 0 h emission spectra. Each emission spectrum obtained upon 320 nm excitation was divided by the 300 nm emission intensity value of the corresponding 0 h emission spectra obtained upon 276 nm excitation. It was not possible to use the 0 h emission spectra recorded at 320 nm for correction since there is emission peak at 0 h for this excitation wavelength. The excitation spectra acquired with emission fixed at 303 nm was divided by the 278 nm excitation intensity values of the corresponding 0 h emission spectra. The excitation spectra obtained with emission fixed at 405 nm were divided by the 278 nm emission intensity value of the corresponding 0 h excitation spectra obtained with emission fixed at 303 nm. Zero h emission and excitation spectra obtained before 1 h excitation were normalized using the respective correction factors mentioned above and used for representation of the non-illuminated insulin spectra.

Normalized emission and excitation spectra were obtained by dividing each data point by the maximum intensity value in each spectrum.

#### Time-based fluorescence emission kinetic traces (vs 276 nm exc. time)

Time-based fluorescence emission kinetic traces obtained with emission set at 303 nm and 405 nm, and excitation fixed 276 nm were normalized by dividing each data point by the emission intensity value at 1 h excitation time.

The emission intensity values at 405 nm used for plotting the kinetic trace of fluorescence emission intensity at 405 nm with excitation fixed at 320 nm were obtained directly from the emission spectra recorded upon 320 nm excitation. The time-based fluorescence kinetic trace was then normalized by dividing each data point (emission intensity) by the 1 h excitation emission.

#### CD spectra

All spectra were obtained in mdeg directly from the instrument and further treated in Origin Pro 8.0. The ellipticities of the samples after different excitation periods were all corrected for the buffer contribution. Near-UV CD spectra were smoothed using a 5 points adjacent averaging.

#### Radioimmunoassay and glucose uptake assay

For both assays data was represented as the mean ± standard error of mean.

In the radioimmunoassay the concentration detected for the insulin samples has been normalized in order to compare the two sets of experiments (1.5 and 3.5 h exc. and dark). Normalization was carried out using as reference the non-illuminated PC insulin detected concentration values (individually for each set of experiments, 1.5 h and 3.5 h) and setting these values to 1.

Unpaired student t-tests were used to compare the samples in the glucose uptake assay. The students t-tests were carried out in Origin Pro 8.0. A p-value <0.05 was considered significant.

#### Fitting Procedures

Normalized fluorescence kinetic traces obtained upon 276 nm illumination (emission set at 303 nm and/or 405 nm, excitation set at 276 nm or 320 nm) were fitted with an exponential function *F(t) = C_1_– C_2._e* ^−*kt*^, where *F(t)* is the fluorescence emission intensity at 303 nm or 405 nm (a.u.) at the 276 nm excitation time *t* (h), *C_1_* and *C_2_* are constants and *k* is the rate constant of fluorescence emission intensity increase (h^−1^).

The kinetics of thiol group formation versus 276 nm excitation time have also been fitted according to an exponential function *y = y_0_– A_._e* ^−*R0t*^, where *y* is the concentration of thiol groups (µM) at the 276 nm excitation time *t* (h), *y_0_* and *A* are constants and *R0* is the rate of thiol group formation (h^−1^).

Smoothing procedures, data fitting and plotting were done in Origin Pro 8.0.

## Results

### Three-dimensional Structure of Insulin

In Figure 2A the 3D crystal structures of native pig insulin monomer and dimer in the hexamer 2Zn structure are presented. Human and pig insulin differ just in one amino acid (human: ThrB30, pig: AlaB30), implying that they share the same protein fold and structure. The insulin monomer contains 4 Tyr residues and as previously mentioned 3 SS. The closest distances between Tyr and Cys residues involve Tyr19 A. TyrA19 is ∼5.4 Å away from CysA6-CysA11 and ∼6.9 Å from CysB19-CysA20. In the insulin monomer, no Tyr residue is in direct contact with another Tyr residue. The shortest distance between Tyr residues is ∼7.5 Å (Tyr19A-Tyr26B**)**. In the insulin dimer Tyr26B from one molecule is in direct Van der Vaals contact (≤5.2 Å as defined by Li and Nussinov [45]) with Tyr16B in the second molecule: for TyrB26 (molecule 1) and TyrB16 (molecule 2) distance is ∼3.8 Å, and for TyrB26 (molecule 2) and TyrB16 (molecule 1) distance is ∼4.1 Å (see Figure 2A).

### UV-Absorption (Prior and after 0.25–2.5 h 276 nm exc., Power Fluency of 2.20 W.m^−2^)

**Figure 3:**
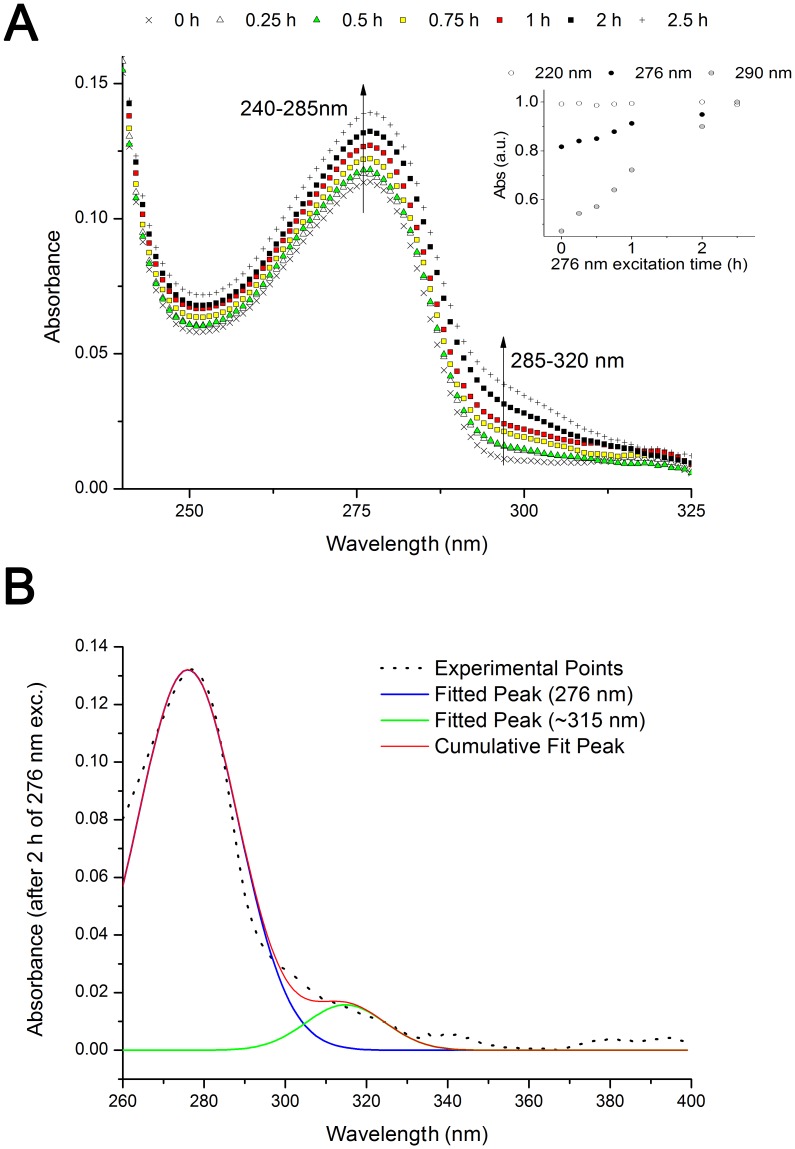
Absorbance spectra of human insulin after prolonged 276 nm UV-excitation. (A) Absorbance spectra obtained before and after 276 nm light continuous exc. (0.25 h, 0.5 h, 0.75 h, 1 h, 2 h, and 2.5 h) of human insulin in solution. In the insert, are plotted the absorbance values at 220, 276 and 290 nm *vs* 276 nm exc. time. Absorbance at 276 and 290 nm increase linearly with 276-nm excitation time (linear fitting, R^2^ = 96.78% for Abs_276 nm_ and R^2^ = 99.28% for Abs_290 nm_). (B) Fitting of the absorbance spectrum (obtained after 2 h exc. with 276 nm light) with a 2 gaussian peak function. The peak at 276 nm was fixed. The original experimental spectrum, the two individual fitting curves obtained for each peak and the cumulative curve of the two fittings are displayed.

Absorbance spectra were acquired prior and after 0.25–2.5 h excitation at 276 nm (Figure 3A). UV-excitation of insulin leads to an increase in absorbance intensity at 240–285 nm, (where Tyr absorbs) and at 285–330 nm. Fitting the absorbance values between 240 nm and 400 nm with a 2-Gaussian peak function shows the presence of two peaks centered at ∼276 nm and at ∼315 nm (Figure 3B and Table 1). The area under each peak tends to increase with UV-excitation time (fitted area, Table 1).

**Table 1:** Parameter values obtained upon fitting of the insulin absorbance spectra with a 2 Gaussian peak function.

Exc. time (276 nm)		λ_max_ (nm)	Fitted area	FWHM (nm)	R^2^
0 h	Peak 1	276	3.070±0.065	25.08±0.66	0.95566
	Peak 2	315.6 ±2.0	0.22±0.050	17.99±4.77	
0.25 h	Peak 1	276	3.259±0.064	25.95±0.64	0.96477
	Peak 2	315.9±1.8	0.262±0.049	19.62±4.31	
0.5 h	Peak 1	276	3.319±0.069	26.11±0.69	0.96078
	Peak 2	315.3±2.2	0.231±0.053	19.62±5.24	
0.75 h	Peak 1	276	3.521±0.068	26.97±0.67	0.96948
	Peak 2	316.5±1.7	0.329±0.053	22.34±4.23	
1 h	Peak 1	276	3.725±0.069	27.50±0.65	0.97289
	Peak 2	314.4±1.3	0.361±0.05	20.04±3.22	
2 h	Peak 1	276	4.089±0.068	29.08±0.63	0.98307
	Peak 2	314.8±1.4	0.387±0.049	23.04±3.29	
2.5 h	Peak 1	276	4.436±0.08	30.26±0.68	0.98496
	Peak 2	316.7±1.5	0.519±0.059	27.72±3.41	

Fitting parameters and corresponding errors (standard error), and root mean square errors were obtained after fitting each insulin absorbance spectrum (spectra in Figure 3A and fitting example in Figure 3B) with a 2 Gaussian peak function. λ_max_ is the wavelength of maximum emission for each absorbance peak. The peak at 276 nm was fixed.

### Steady State Fluorescence Emission (Prior and after 0.5–7 h 276 nm exc., Power Fluency of 2.20 W.m^−2^)

#### Emission Spectra (excitation 276 nm)

**Figure 4:**
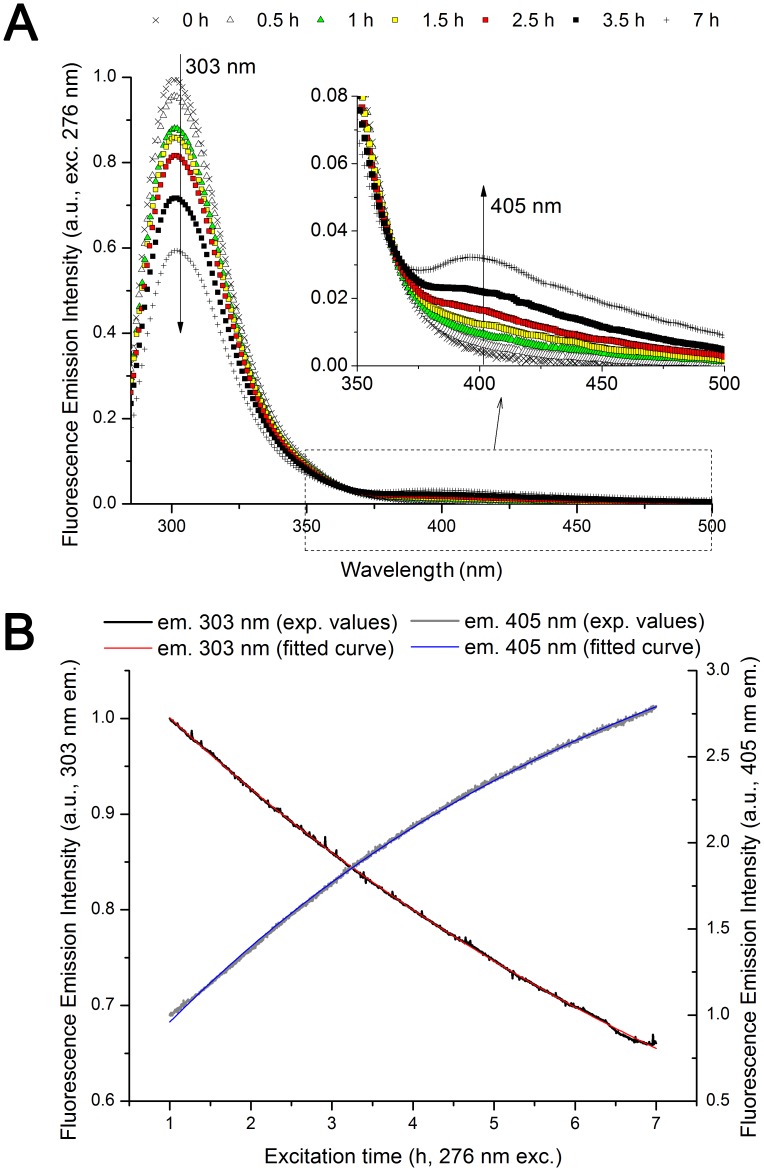
Fluorescence emission of human insulin (276 nm excitation) upon prolonged 276 nm UV-excitation. (A) Fluorescence emission spectra (276 nm exc.) obtained before and after 276 nm light continuous exc. (0.5 h, 1 h, 1.5 h, 2.5 h, 3.5 h, and 7 h) of human insulin in solution. There is a continuous decrease in emission intensity at 303 nm with 276 nm exc. time. The insert shows a zoom of the emission spectra between 350 and 550 nm. Emission intensity at ∼405 nm increases progressively with 276 nm excitation time. (B) Fluorescence emission intensity kinetic traces obtained at 330 and 405 nm (exc. fixed at 276 nm) upon continuous of human insulin with 276 nm light. Fitting of the experimental traces was carried out using an exponential function *F(t) = C_1_– C_2._e* ^−*kt*^. Fitted parameter values and corresponding errors, and root mean square error values were obtained after fitting each kinetic trace (Table 2).

In Figure 4A is shown the emission spectra of insulin acquired upon excitation at 276 nm (absorption maximum wavelength for Tyr) prior and after 276 nm excitation. A pronounced decrease in emission intensity (centered at 301 nm) is observed after UV-excitation. After 7 h continuous 276 nm excitation the emission intensity of the insulin tyrosine residues at 303 nm is only ∼60% of the initial fluorescence emission intensity. The observed decrease in emission intensity at 303 nm is correlated with an increase in fluorescence emission intensity at 405 nm (Figure 4A, insert). After 7 h of excitation a peak is clearly visible at ∼405 nm and a 6.2 fold intensity increase is observed. Normalization of the emission spectra (data not shown) shows no shift in the wavelength of the most intense peak centered at 301 nm. The change in fluorescence emission intensity at 303 nm and 405 nm upon prolonged 276 nm excitation time is displayed in Figure 4B. Fitting the experimental curves show that both changes are exponential. The results are listed in the section below.

**Table 2:** Parameter values obtained upon fitting the insulin fluorescence emission kinetic traces recorded with emission fixed at 303 nm (exc. 276 nm) and 405 nm (exc. 276 or 320 nm).

Fitting Fig.	Exc. (nm)	Em. (nm)	*C_1_*	*C_2_*	*k* (h^−1^)	R^2^	
**Fig. 4B**	276	303	0.277±0.002	−0.806±0.002	1.08E−01±4.04E−04		0.99979
	276	405	3.935±0.007	3.489±0.006	1.59E−01±6.05E−04		0.99955
**Fig. 5B**	320	405	9.191±0.161	9.622±0.135	1.62E−01±5.08E−03		0.99992

**Figure 5:**
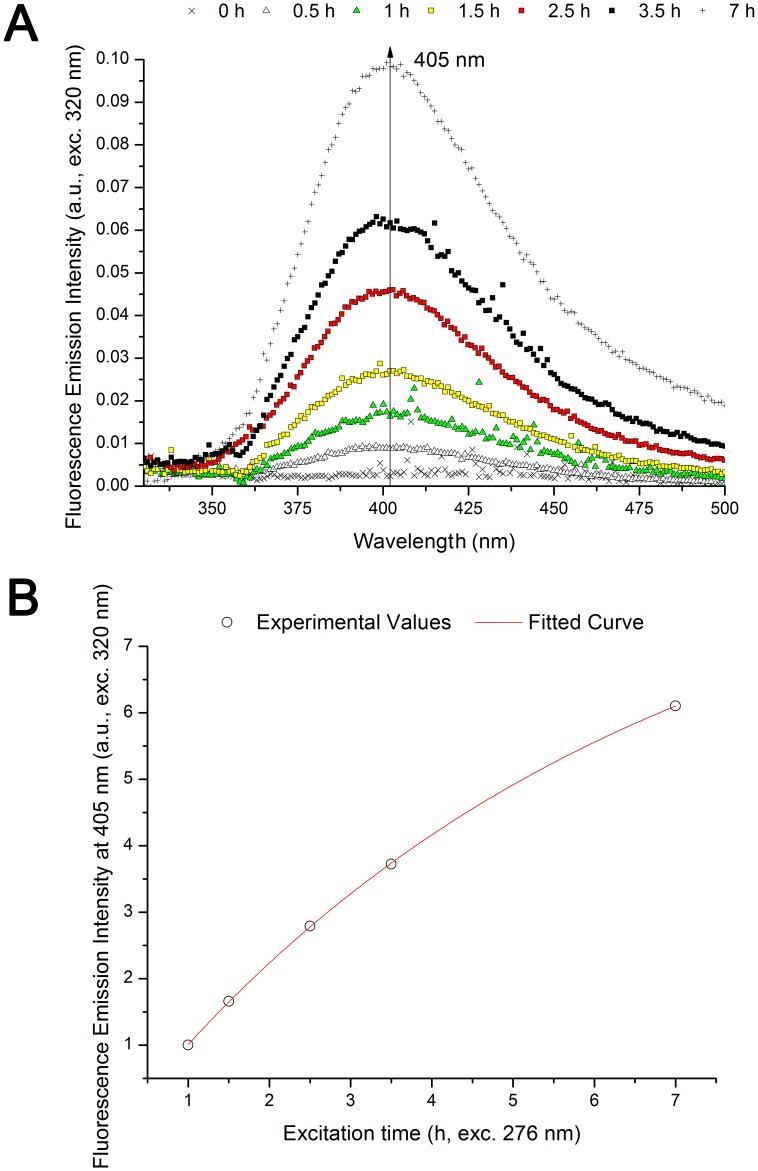
Fluorescence emission of human insulin (320 nm excitation) upon prolonged 276 nm UV-excitation. (A) Fluorescence emission spectra (320 nm exc.) recorded before and after 276 nm light continuous exc. (0.5 h, 1 h, 1.5 h, 2.5 h, 3.5 h, and 7 h) of human insulin in solution. There is a continuous increase in emission intensity at 405 nm with 276 nm exc. time. (B) Fluorescence emission intensity kinetic trace obtained at 405 nm (exc. at 320 nm) upon continuous of human insulin with 276 nm light. Fitting of the experimental traces was carried out using an exponential function *F(t) = C_1_– C_2._e* ^−*kt*^. Fitted parameter values and corresponding errors, and root mean square error values were obtained after fitting each kinetic trace (Table 2).

Fitting parameter values and corresponding errors (standard errors), and root mean square errors were calculated after fitting the fluorescence emission kinetic traces recorded with emission fixed at 303 nm (exc. fixed at 276 nm, Figure 4B) and 405 nm (exc. fixed at 276 nm and 405 nm, Figures 4B and 5B) upon continuous 276 nm excitation to the equation *F(t) = C_1_– C_2._e* ^−*kt*^.

#### Emission spectra (excitation 320 nm)

After exciting insulin with 276 nm light, emission spectrum of the protein were recorded upon 320 nm excitation in order to verify if the species that absorbs at these wavelengths is also fluorescent (Figure 5A). Before 276 nm excitation, no fluorescence emission is observed at 330–500 nm upon 320 nm excitation. With the increase in 276 nm excitation time, we observe the progressive formation of a peak centered at ∼400 nm (exc. 320 nm). After 7 h of excitation, the emission intensity at 405 nm is 31.4 fold higher compared to non-illuminated insulin. The kinetics of the fluorescence emission intensity increase at 405 nm versus 276 nm exc. time is exponential (Figure 5B and Table 2). In Table 2 are summarized the fitted parameters and root mean square errors obtained upon fitting the kinetic traces displayed in Figures 4B and 5B with an exponential model (*F(t) = C_1_– C_2._e* ^−*kt*^). After prolonged excitation with 276 nm light, there is an exponential increase of fluorescence emission intensity at 405 nm (Figures 4B and 5B) and an exponential decrease of fluorescence emission intensity at 303 nm (Figure 4B) upon 276 nm excitation. The kinetics traces have been fitted in the same time window (1–7 h) and normalized to the emission value at 1 h of 276 nm excitation.

In Table 2 we observe that the kinetics of emission intensity increase at 405 nm upon 276 nm or 320 nm excitation are similar with comparable kinetic constant *k* (constant that determines exponential fluorescence emission increase over 276 nm exc. time) values (1.59.10^−1^ h^−1^, exc. 276 nm and 1.62.10^−1^ h^−1^, exc. 320 nm). However, the fluorescence emission intensity increase at 405 nm upon 320 nm excitation is faster (higher *C_2_* value). The kinetics of fluorescence emission intensity decrease at 303 nm (exc. 276 nm) are distinct and slower, with a lower *k* value (1.08.10^−1^ h^−1^).

#### Excitation spectra (emission 303 nm)

**Figure 6:**
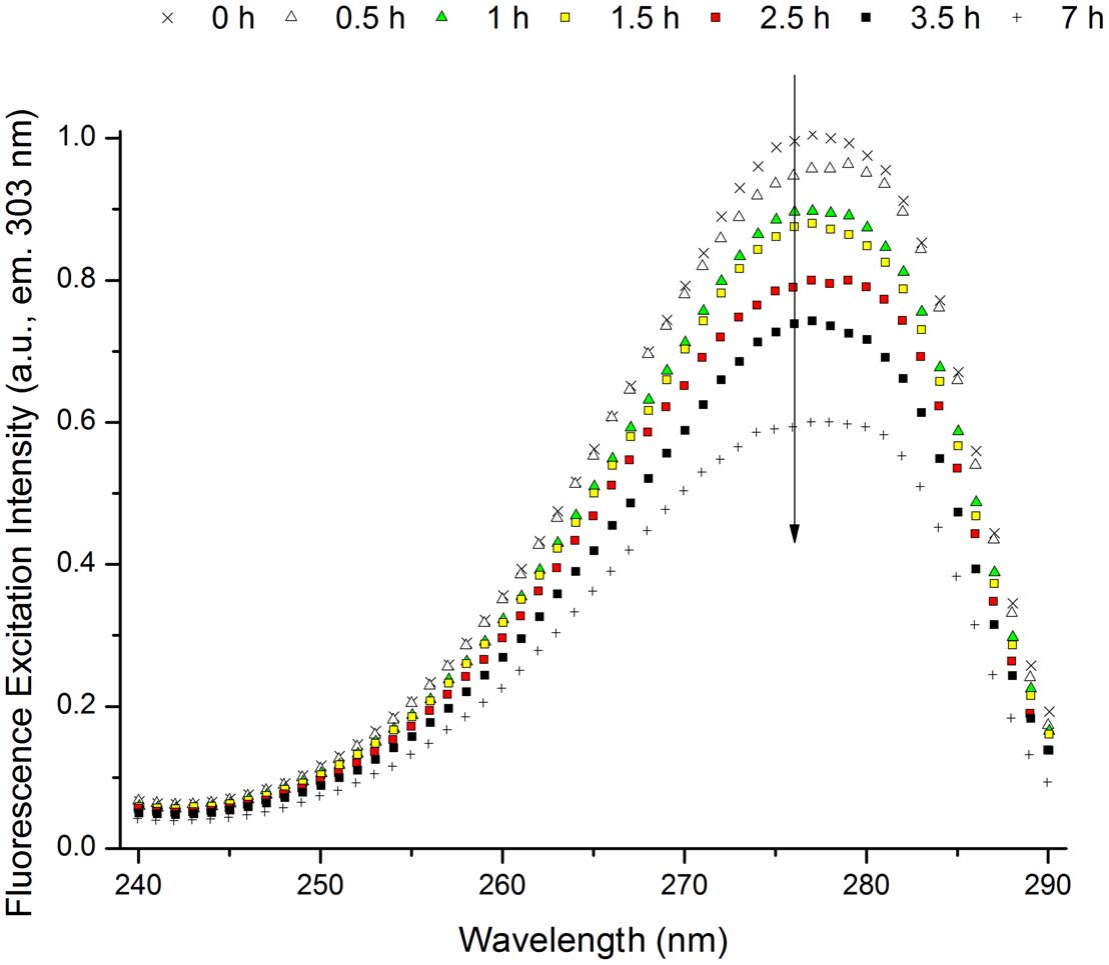
Fluorescence excitation spectra of human insulin (emission fixed at 303 nm) upon prolonged 276 nm UV-excitation. The excitation spectra (em. 303 nm) were obtained before and after 276 nm light continuous exc. (0.5 h, 1 h, 1.5 h, 2.5 h, 3.5 h, and 7 h) of human insulin in solution. There is a continuous decrease in excitation intensity at 276 nm with 276 nm exc. time.

In Figure 6 is shown the excitation spectra of insulin acquired by fixing the emission at 303 nm, where Tyr should emit, prior and after 276 nm excitation. It can be observed that the excitation emission intensity decreases over the illumination time, which is correlated with the previously described decrease in emission intensity at 303 nm (Figure 4A). Normalization of the excitation spectra shows no shift in the wavelength where maximum excitation intensity is observed (∼277 nm), matching the absorption maximum of Tyr (data not shown).

#### Excitation spectra (emission 405 nm)

**Figure 7:**
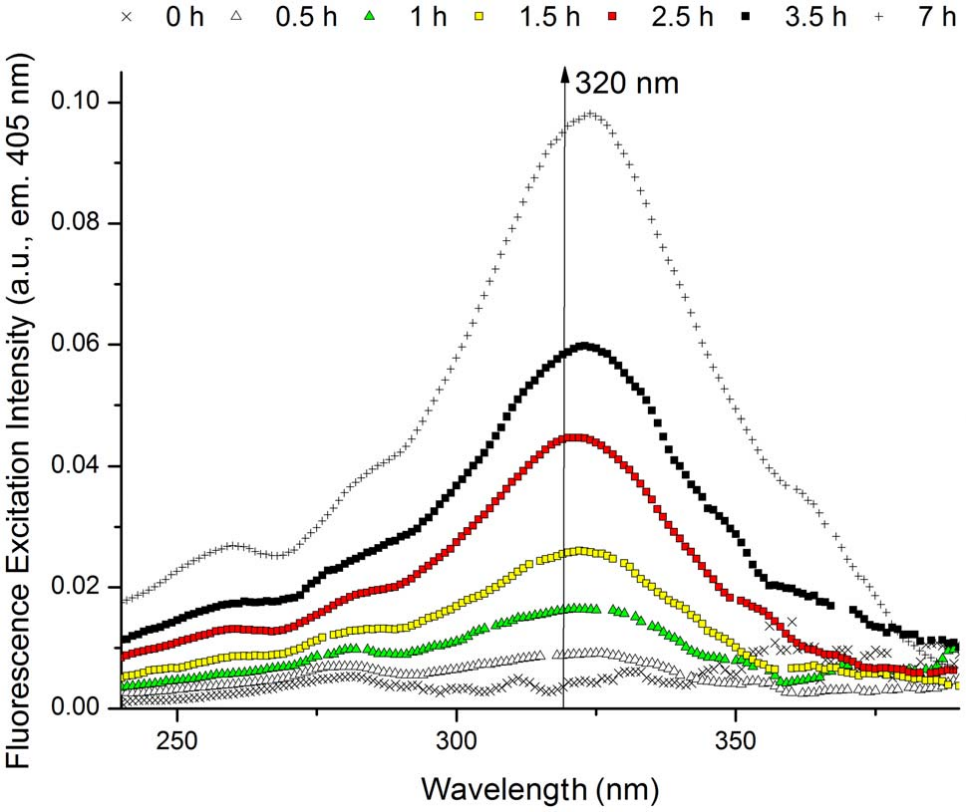
Fluorescence excitation spectra of human insulin (emission fixed at 405 nm) upon prolonged 276 nm UV-excitation. Fluorescence excitation spectra (emission fixed at 405 nm) obtained before and after 276 nm light continuous exc. (0.5 h, 1 h, 1.5 h, 2.5 h, 3.5 h, and 7 h) of human insulin in solution. There is a continuous increase in excitation intensity at 320 nm with 276 nm exc. time.

In order to analyze the species that contribute to the increase of fluorescence emission intensity at 405 nm after continuous 276 nm excitation, excitation spectra (emission fixed at 405 nm) were acquired after each continuous excitation session (Figure 7). Upon 276 nm excitation, a progressive increase in the excitation intensity centered around 320–325 nm is observed. After 7 h, excitation intensity increases ∼22 fold at 320 nm. Normalization of the excitation spectra shows no shift with excitation time on the peak formed at ∼320–325 nm (data not shown).

### Thiol group’s quantification (Prior and after 0.25–2.5 h 276 nm exc., Power Fluency of 2.20 W.m^−2^)

**Figure 8:**
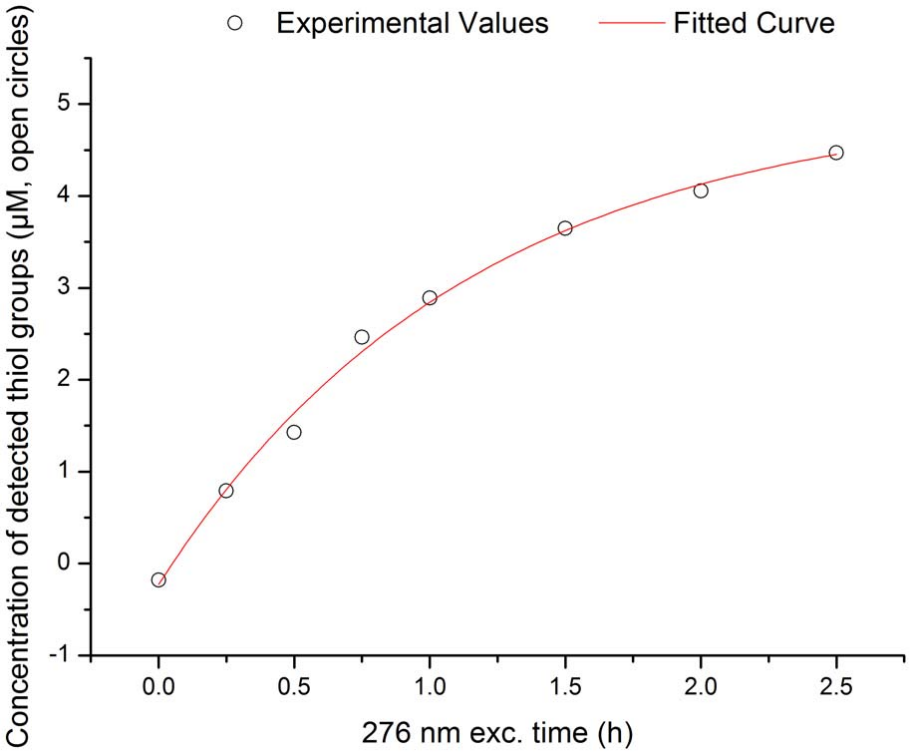
Concentration of detected free thiol groups (open circles) in human insulin *vs* 276 nm exc. time. Detection of free thiol groups was carried out using the Ellman’s assay before and after 276 nm light continuous exc. (0.25 h, 0.5 h, 0.75 h, 1 h, 2 h, and 2.5 h) of human insulin in solution. The concentration of free thiol groups was estimated from the absorbance of the Ellman’s assay reaction product, TNB^2−^, at 412 nm (ε_412 nm_ = 14150 M^−1^.cm^−1^ [44]). The experimental values were fitted using an exponential function *y = y_0_– A_._e* ^−*R0t*^ (fitted curve in red), where *y* is the concentration of thiol groups (µM) at the 276 nm excitation time *t* (h), *y_0_* and *A* are constants and *R0* is the rate of thiol group formation (h^−1^). Fitted experimental parameters were: *y_0_* = 5.04±0.24 µM, *A* = 5.27±0.22 µM, *R0* = 0.87±0.09 h^−1^. Root mean square error was 99.41%.

The concentration of free thiol groups increases with 276 nm excitation time, following an exponential kinetic (Figure 8). After 2.5 h the concentration of free thiol groups in insulin is ∼4.47 µM. Assuming that the formation of free thiol groups follows a first order kinetics (as indicates the 1^st^ order exponential model used) it is possible that more thiol groups are formed with increased 276 nm excitation time. The maximum value of thiol concentration can be estimated from the exponential model (*y = y_0_– A_._e* ^−*R0t*^) used for fitting and is given by *y_0_*, which is of 5.04 µM.

### CD measurements (Prior and after 0.5 h –7 h 276 nm exc., Power Fluency of 2.20 W.m^−2^)

**Figure 9:**
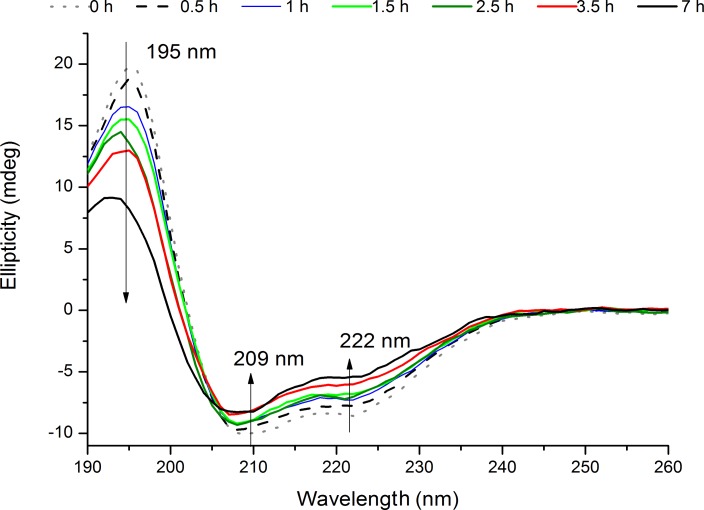
Far-UV CD spectra of human insulin recorded before upon prolonged 276 nm UV-excitation. The far-UV CD spectra were obtained before and after 276 nm light continuous exc. (0.5 h, 1 h, 1.5 h, 2.5 h, 3.5 h, and 7 h) of insulin in solution. There is a progressive loss of ellipticity signal with 276 nm exc. time at 195, 209 and 222 nm.

The far-UV CD spectrum of the non-illuminated sample (exc. 0 h) displays the classical far-UV features characterizing protein secondary structure (Figure 9). There is a peak at ∼195 nm, representative of β-sheet organization and a double minimum at 222 nm and 210–208 nm, characteristic of α-helical content [46]. Prolonged excitation with 276 nm light leads to a progressive decrease of ellipticity at 195, 210–208, and 222 nm (Figure 9). After 7 h of illumination, there is a decrease of 58.5%, 18.1% and 37.15% of ellipticity signal at 195, 209 and 202 nm, respectively.

Prolonged excitation of insulin with 276 nm light results in a loss in near-UV CD ellipticity signal. After 3.5 h of 276 nm excitation there is a 66.2% decrease ellipticity signal at 273 nm (data not shown).

### Radioimmunoassay (1.5 h, 3.5 h 276 nm exc., Power Fluency of 2.20 W.m^−2^)

**Figure 10:**
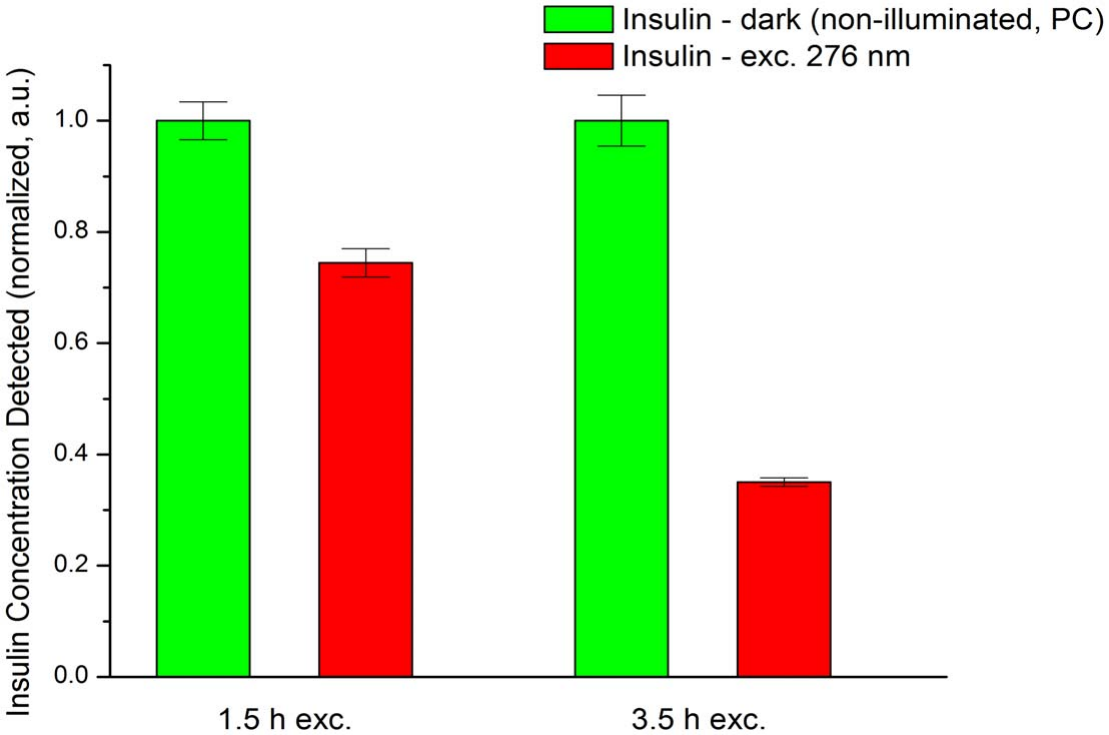
Effect of continuous 276 nm light exposure of human insulin on its recognition by guinea-pig anti-insulin antibodies. Human insulin concentration was detected using a radioimmunoassay. Insulin samples were previously excited with 276 nm light during 1.5 and 3.5 h. A positive control (PC) was carried out for each excitation duration, where insulin was left in the dark for the same time period (1.5 and 3.5 h). Uncertainty values (standard errors) for the detected insulin concentration are displayed with error bars.

Continuous UV-excitation leads to a progressive decrease in insulin concentration detected by the guinea-pig anti-porcine insulin antibodies (Figure 10). After 1.5 h of illumination the detected insulin concentration is 25.6% lower than the one for the positive control sample (non-illuminated insulin, PC). After 3.5 h of excitation, the concentration of insulin molecules detected was 65.0% lower than for the PC sample.

### Glucose Uptake Assays (1.5 h 276 nm exc., Power Fluency of 2.20 W.m^−2^)

**Figure 11:**
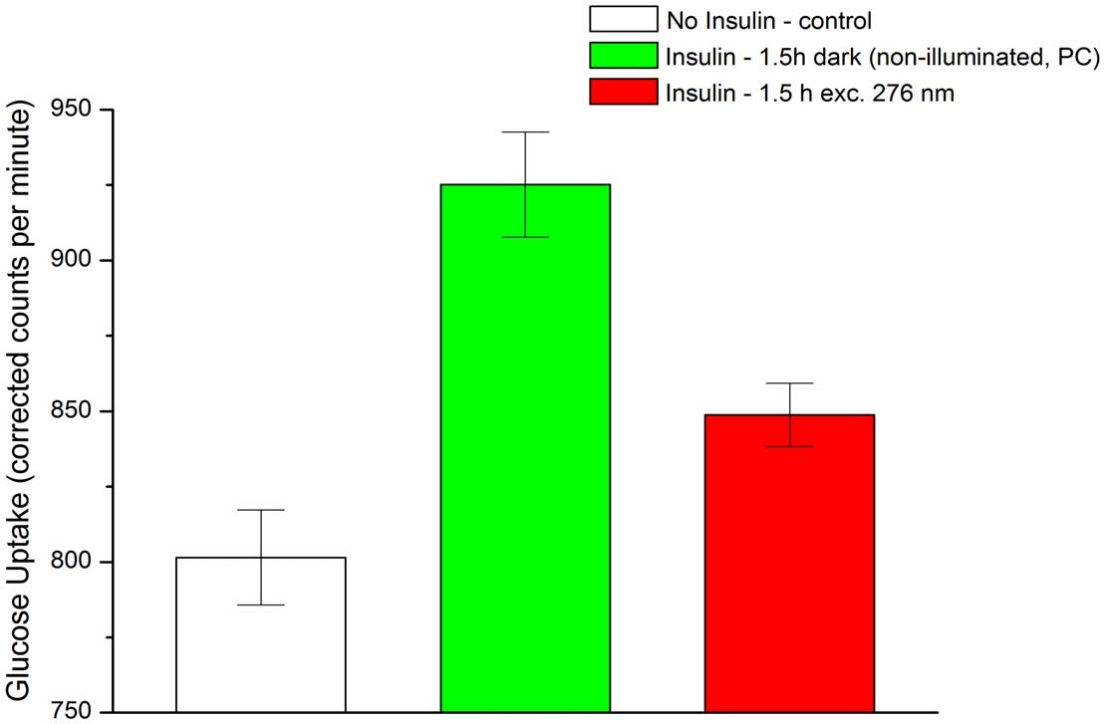
Effect of continuous 276 nm light exposure of human insulin on its hormonal function *in vitro*. The glucose uptake by human skeletal muscle cells was measured after 1 hour of incubation at three different conditions: no insulin (control), insulin kept in the dark (positive control, PC), and UV-illuminated insulin (1.5 h exc. at 276 nm). Uncertainty values (standard errors) for the concentration of glucose (uptake) are displayed with error bars. The students t-tests resulted in the following p-values: p (control *vs* PC) = 27.6.10^−4^; p (control *vs* 1.5 h exc. at 276 nm) = 0.002165; p(PC *vs* 1.5 h exc. at 276 nm) = 0.00179.

Figure 11 shows the glucose uptake (counts per minute) by skeletal muscle cells after 1 hour of incubation at three different conditions: no insulin, insulin kept in the dark (positive control, PC), and pre-treated UV-illuminated insulin (1.5 h excitation at 276 nm). As expected, there is a considerable increase in glucose uptake (15.4±4.1%) for the cells that have been stimulated with insulin (non-illuminated, positive control, PC) when compared to the cells void of insulin (p<0.05). When the cells were stimulated with the same concentration of insulin previously illuminated with 276 nm light, the increase in glucose uptake relative to the cells void of insulin is 5.9±3.3% (p<0.05). The difference in glucose uptake between the positive control samples (PC) and the excited samples (1.5 h excitation at 276 nm) is significant (p<0.05). After 1.5 h of excitation with 276 nm light, insulin retains only 38.3% of its normal activity *in vitro*. The same experiment was repeated in similar conditions yielding comparable results (data not shown). Such data shows that stimulation of cells with non-illuminated and 1.5 h 276 nm excited insulin resulted respectively in 16.2±7.2% and 3.3±6.1% increase in glucose uptake. Only 20.2% of the insulin hormonal function was retained after exposure with UV-light.

## Discussion

The response of insulin molecules to UV-light and the interaction between illuminated insulin molecules will depend greatly on which form insulin will be in solution (monomer, dimer, or higher order aggregational states). In our experiments, human insulin is present at a 17.7 µM concentration. Currently a widely accepted model for concentration-dependent association of insulin in zinc-free solutions is the one of Pekar and Frank [47]. It assumes that two insulin monomers form a dimer, three dimers form a hexamer, and subsequently self-association proceeds with addition of hexamers (the model does not consider tetramer formation). Using the equilibrium constants determined by Attri et al. (2010) [40] for insulin self-association (20 mM Phosphate, 0.1 M NaCl, 5 mM EDTA, pH 8.0) in the model for a concentration of 17.7 µM we obtain relative populations of monomer, dimer, and hexamer of 1.66%, 97.75% and 0.59%, respectively (we excluded aggregation states of higher order than hexamer). Thus, it is expected that in our experiments, insulin is mainly present in its dimeric form.

The structural interaction between the insulin peptide and its membrane receptor is a key process in the biological and physiological action of the hormone. It is well established that insulin molecules bind to its receptor in its monomeric form, but the mechanisms of binding are not completely understood. The classical receptor binding surface in insulin is constituted by a number of residues: GlyA1, GlnA5, TyrA19, AsnA21, ValB12, TyrB16, GlyB23, PheB24, PheB25 and TyrB26 (Figure 2A, CPK in blue) [38], [48]. For convenience we will refer this binding surface as binding site 1. Furthermore, it has been shown that the N-terminal α-helix of the insulin A chain (amino acids A1-A8) is relevant for achieving high affinity insulin receptor activation for insulin recognition [38], [48]. A second cluster of residues has been proposed to be important for insulin receptor binding: SerA12, LeuA13, GluA17, HisB10, GluB13 and LeuB17 (Figure 2A, residues displayed as CPK in red) [38]. We will name this second binding region as binding site 2. It is proposed that these two separate binding zones in insulin allow the interaction with two separate binding sites in the insulin receptor [38], [49].

In its aggregated forms insulin is inactive and does not bind to the insulin receptor [50]. In fact, in the physiologically present aggregated forms of insulin (dimer conformation and 2Zn hexamer), several residues relevant for insulin receptor binding (sites 1 and 2) are involved in dimer interaction and thus blocked from insulin receptor recognition (see Figure 2B). In the insulin dimer conformation (Figure 2A) the key interactions between insulin molecules 1 and 2 are complementary (anti-symmetric) and involve hydrogen bonding between residues of the classical receptor binding site 1: TyrB26 of one molecule and PheB24 of the other molecule [50], [51]. Other important residues of the classical receptor binding site 1 that also participate in dimerization are PheB25, TyrA19 and TyrB16 [50], [51]. Upon dimer formation, almost all the residues of the receptor binding site 1 are either blocked or solvent shielded (Figure 2B, in blue). It is also relevant to mention that Tyr A14 is involved in dimer interaction to form the insulin 2Zn hexamer [35], and that 5 of the 6 residues of the binding site 2 are either blocked or solvent shielded (Figure 2B, in red). In the case of permanent dimerization of the insulin dimer it is expected that some of the binding site 1 protein residues remain inaccessible for receptor recognition, which would result in permanent loss of insulin functionality.

The changes in fluorescence emission of insulin induced by continuous UV-excitation concur with the progressive formation of a new species that emits strongly at ∼405 nm (Figure 4A). This species is excited at 276 nm and more strongly at 320 nm (Figure 7), which correlates well with the fluorescence characteristics of dityrosine formed upon cross-linking of two tyrosine molecules (*vide supra*, introduction). The progressive increase in excitation intensity at 276 and 320 nm upon continuous 276 nm excitation also confirms that new dityrosine molecules are constantly formed upon UV-excitation. Dityrosine formation is also correlated with the observed increase in absorption of insulin at 276 and 314 nm upon UV-excitation (Figures 3A, 3B and Table 1). The two fitted absorption peaks correspond to the absorption maxima of dityrosine species, at 284 and 315 nm (vide supra, *introduction*). Tyrosil radicals may also be involved in other reactions than dityrosine coupling. Some of the tyrosil radicals disproportionate (reaction between radicals to originate non-radical products) to tyrosine and dihydroxyphenylalanine (DOPA) [19], [20]. Furthermore, other products of tyrosine oxidation may be present such as isodityrosine, trityrosine, pulcherosine, dopamine, dopamine quinone, 5,6-dihydroxyindol and 5,6-dihydroxy-3-oxo-indol [12], [20]. The yields of formation of these products are usually lower compared to dityrosine [12].

Dityrosine formation has been observed previously in insulin upon oxidation with H_2_O_2_/peroxidase [15], metal-catalyzed oxidation with H_2_O_2_/Cu [52], ozone [53], carbon electrodes [54], and exposure to γ-radiation [17]. Inter-molecular dityrosine cross-linking is indicated by two of these studies [15], [52], while carbon electrode oxidation suggests intra-molecular dityrosine cross-linking of insulin [54]. The minimum distance between tyrosine residues within an insulin monomer is 7.5 Å which does not favor direct Van der Vaals contacts (Results and Figure 2A). Thus, it is more likely that tyrosine cross-linking occurs between two different insulin molecules. In the dimer conformation (Figure 2A), Tyr26B from one molecule is in direct Van der Vaals contact (≤5.2 Å [45]) with Tyr16B in the second molecule and *vice-versa*. These two residue pairs are also involved in the dimer stabilization via hydrogen bonding. This geometry favors direct dityrosine cross-linking, indicating that excitation of insulin with UV-light leads to progressive covalent cross-linking of the insulin molecules in the dimer. In the dityrosine insulin dimer the classical binding region would not be available for receptor recognition, resulting in permanent loss of function, as observed in our study (Figure 10). Since the tyrosil radical Tyr^•^ is relatively stable, with a lifetime in the microsecond timescale [2], we cannot exclude that the cross-linking occurs differently between insulin molecules of different dimers.

The fluorescence emission intensity at 405 nm upon insulin 276 nm prolonged illumination follows a single exponential increase for excitation wavelength either at 276 nm (Figure 4B and Table 2) or 320 nm (Figure 5B and Table 2). The fitted constant *k* (constant for exponential fluorescence emission intensity increase) is similar at the two excitation wavelengths which indicate that it is the same process that occurs in both cases. The two excitation wavelengths (276 nm and 320 nm) can excite separately the protonated and single deprotonated forms of dityrosine (absorption maxima of 284 and 314 nm, respectively, *vide supra* introduction). Upon excitation to its singlet excited states, both of these species fluoresce at ∼400–409 nm. This can be interpreted as if dityrosine is formed upon UV-excitation of tyrosine following a 1^st^ order reaction, and that upon continuous excitation of its protonated and single deprotonated forms, dityrosine fluoresces at 405 nm. This is in agreement with the photochemical reaction mechanism for dityrosine cross-linking from tyrosine radical described previously (*vide supra*, introduction, Figure 1A).

A new molecular species is formed upon UV excitation of insulin: dityrosine. Simultaneously with the formation of dityrosine, tyrosines must disappear. Upon prolonged illumination at 276 nm, tyrosine emission at 303 nm diminishes (Figure 4A) Furthermore, tyrosine excitation intensity at 276 nm (303 nm em., Figure 6) decreases. This contrasts with the already mentioned decrease of 276 nm absorbance with excitation time (Figure 3A). At 276 nm the extinction coefficient of dityrosine is higher than the summed extinction coefficients of the forming two tyrosine molecules (at 276 nm – tyrosine, extinction coefficient of 1362 cm^−1^.M^−1^ [7]; dityrosine extinction coefficient of 3068 cm^−1^.M^−1^ (extracted from [55]). Since dityrosine is also progressively formed upon continuous UV-excitation, the replacement of tyrosine molecules with dityrosine will lead to an increase of absorption intensity at 276 nm. The formation of other tyrosine derivatives upon UV-excitation (please see discussion above) may also contribute to the increase in absorption intensity at 276 nm, (e.g. for DOPA: ε_280 nm_ = 2692 M^−1^.cm^−1^,in buffer, pH 5.6 and 6.8 [56]). The decrease in fluorescence emission intensity at 303 nm follows a single exponential decay kinetics upon continuous 276 nm excitation (Figure 4B). The exponential model is consistent once more with a 1^st^ order reaction, which is in agreement with the mechanisms that precede tyrosine depletion, such as Tyr photoionization and intersystem crossing to the triplet state.

The depletion of tyrosine molecules and formation of dityrosine cross-linking with UV-light is correlated with growing amounts of free thiol (SH) groups with the Ellman’s assay (Figure 8). This is consistent with the breakage of the protein’s SS bonds upon UV-excitation of tyrosine, a well-described mechanism in proteins [2], [3], [27], [28]. As previously observed (*vide supra* results – *three-dimensional structure of insulin*) in the insulin monomer (Figure 2A) no Tyr residue is in direct Van der Vaals contact with SS bonds. Nonetheless, TyrA19 is close to CysA6-CysA11 (∼5.9 Å) and CysB19-CysA20 (∼6.5 Å), which would favor direct electron transfer to these residues after formation of ^3^Tyr (*vide supra* – introduction, scheme 4). Photoionization of Tyr and formation of solvated e^−^_aq_ may also be a mechanism involved in breakage of SS in insulin, which could be the case for longer distances between Tyr and SS (*vide supra* – Introduction, schemes 1 and 2). The formation of free SH groups upon continuous 276 nm excitation of insulin follows a single exponential kinetics which would indicate that the breakage of SS bonds is consistent with a first order reaction. The maximum value of free SH concentration is 5.04 µM. Considering that the protein concentration in the experiments is 17.7 µM and that insulin has 3 SS, the maximum concentration of free thiol groups that could be present would be of 106.2 µM. Thus, we conclude that at least one SS in insulin has been disrupted in the insulin molecules after 276 nm excitation. The number of SS broken may be higher than the estimated by the Ellman’s assay.

Moziconacci et al. reported UV light (253.7 nm) induced photolysis of the SS bonds in human insulin [57]. At this wavelength photolysis of SS occurs both via direct cystine excitation and via tyrosine or phenylalanine excitation. Mozziconaci et al. report that 67% of the thiols formed were generated through direct homolysis of the SS and 33% through solvated electrons yielded from tyrosine and phenylalanine excitation [57]. In total, all of the 3 SS in insulin were prompt to cleavage upon insulin continuous 253.7 nm excitation. The authors reported the existence of different photo-products with SS bonds broken upon 253.7 nm excitation [57]. More relevantly, one of the photo-products yielded from Tyr excitation had a cross-link between TyrA19 and CysB19 resulting from CysB19-CysA20 breakage. The breakage of this CysB19-CysA20 upon TyrA19 excitation is in agreement with our previous observation on the proximity between TyrA19 and this SS. Breakage of SS CysB7-CysB19, and the cross-link TyrA19-CysB19 were also observed in insulin after 253.7 nm excitation. It is likely that breakage of these SS bonds and the above mentioned cross-link also occur in our experiments. TyrA19 belongs to binding site 1. If it is cross-linked with CysB19 it might be blocked and not be able to participate in receptor binding.

Disruption of CysA7-CysB7 and/or CysA20-CysB19 may result in the rearrangement of the native interactions between the A and B chains and the native structural secondary organization. Electrolytic reduction of the intrachain disulfide bonds of crystalline beef zinc-insulin results in losses of α-helix and β-sheet organization content [58]. Dithiothreitol (DTT) reduction of the three SS leads to a conformational transition in insulin, thermodynamically of the same nature as in the thermal denaturation of globular proteins [59]. The CD results described in our study confirm that continuous UV-excitation of insulin leads to loss of secondary structure. The losses in secondary structural content of the protein (Figure 9) can result from breakage of SS, leading to a gradual unfolding of insulin. The major decrease in ellipticity is consistent with a loss of β-content upon UV-excitation. Curiously, the β-strand of insulin is located in the region of dimer interaction. Upon dityrosine cross-linking between the two monomers (through the two tyrosine pairs Tyr26B and Tyr16B), it is possible that some of the native fold is rearranged in this region, resulting in a structural rearrangement of the antiparallel β-sheet. The dramatic losses in near-UV CD can be explained by the rearrangement of the native interactions of the aromatic residues in insulin.

The structural modifications resulting from UV-excitation are correlated with the observed loss in insulin detection by specific insulin antibodies and in the function of insulin as a hormone. The number of insulin molecules detected by the insulin antibodies decreases progressively with the 276 nm excitation time of the insulin solution. After 1.5 h and 3.5 h of excitation the concentration of insulin molecules detected decreases by 25.6% and 65.0%, respectively (Figure 9). It implies that UV-light excitation induces 3D structural changes in insulin, which impair the binding of the insulin antibodies to the peptide hormone.

The antibody serum used in this work for radioimmuno-detection consists on a mixture of polyclonal antibodies prepared by immunization of guinea pigs with porcine insulin. The specificity of this polyclonal mixture has been tested positively for human insulin. Since we are using a polyclonal antibody serum it is expected that separate regions of the insulin molecule can be used as attachment sites for the different insulin antibodies present in the serum [60], [61]. In human insulin epitopes involving the residues GluA4, ThrA8, SerA9, IleA10, AsnB3, ProB28, LysB29 and ThrB30 have been indicated as possible antigenic sites [60]. The insulin antibodies are added as an excess to the insulin in the radioimmunoassay. Thus, it is expected that if there are any structural changes in a single epitope, insulin can still be detected, because antibody molecules could still bind to alternative epitopes/sites would still be plentiful. The insulin molecules that are not detected by the insulin antibodies have probably suffered structural modifications in all or almost all of the epitopes recognized by the antibodies. In that context several regions of the non-detected insulin molecules must have lost their native conformation upon continuous UV-excitation. Within the amino acid residues reported to be part of likely antibody binding sites, Glu4 is part of an α-helix and ThrA8, SerA9 and IleA10 are part of a loop between the 2 α-helices in the A chain of insulin. Any loss of structure nearby these residues is likely to distort the 2 A chain α-helices. This is positively correlated with the decrease in α-ellipticity reported by our far-UV CD studies. Residues ProB28 and LysB29 are involved in the association of 2 insulin molecules into a dimer (see Figure 2B, in blue). If these residues cannot be recognized by the insulin antibodies it may indicate that these insulin molecules have covalently dimerized via dityrosine cross-linking as we will discuss ahead. The glucose uptake assay allows monitoring the efficiency of insulin binding to the insulin receptor, which occurs through specific binding sites. In this case it is easier to identify the structurally modified regions in the insulin molecule upon UV excitation. Glucose uptake has been reduced 61.7% when stimulation has been done with insulin previously excited during 1.5 h with 276 nm (Figure 10). This implies that 1.5 h of UV illumination has induced 3D structural changes in insulin which impaired binding to the receptor. After 1.5 h of 276 nm excitation the number of insulin molecules that are impaired for insulin receptor recognition is quite higher than the number of insulin molecules impaired for antibody recognition (61.7% decrease in glucose uptake *vs* 25.6% decrease in detected insulin by antibodies). After this UV-excitation period, part of the pool of insulin molecules may have suffered structural modifications in the binding sites regions but can still be recognized by the polyclonal antibodies due to the presence of several epitopes. In the radioimmunoassay and glucose uptake assays the insulin used for the experiments (illuminated or not) has been diluted down respectively to 0.0354 and 100 nM concentrations to allow receptor binding to monomeric insulin. During 1.5 h of continuous 276 nm excitation dityrosine has been formed, leading to likely insulin dimerization (Figure 1B). Upon dilution of the illuminated solution down to 0.0354 and 100 nM any dityrosine cross-linked dimers will remain as dimers. As previously discussed, covalent insulin dimerization would lead to loss of the binding capability and consequently loss of functional insulin, which is the monomeric form of insulin. As previously mentioned, metal-catalyzed oxidation of insulin with H_2_O_2_/Cu leads to the formation of dityrosine [52]. In this case, formation of dityrosine is correlated with the formation of insulin aggregates (probably resultant from inter-molecular cross-linking) and loss of the peptide biological function [52]. According to the kinetics of fluorescence emission increase at 405 nm not even half of the dityrosine molecules are formed after 7 h of continuous excitation at 276 nm (Figure 5B, Table 2). As previously mentioned, the yield of dityrosine formation upon UV-excitation of proteins is rather low [20]. Thus, there is/are probably other population(s) of molecules whose native structural fold has suffered conformational changes via other mechanisms, such as SS disruption. After 1.5 h of 276 nm excitation the concentration of SH groups formed is 71.7% of the maximum detected concentration (Figure 8). It means that a considerable number of insulin molecules have had their SS disrupted. The integrity of the three native SS of insulin is essential for the hormone’s biological activity. Disruption or rearrangement of any of these SS results in low or absent bioactivity [48]. Substitution of CysA6 and CysA11 in insulin with a serine or alanine pair reduces the binding affinity to the receptor to 0.1 and 5% of native insulin [48]. Other analogs with group substitutions of CysA7 or CysB7 showed also lower bioactivity than native insulin [48]. Therefore, considering these two major structural modifications it is not surprising that 25.6% of the insulin molecules are no longer detected by polyclonal antibodies and that ∼61.7% have lost their function after 1.5 h of UV-excitation.

### Conclusions

The present study advances conclusions that are of global importance to the shelf life of pharmaceutical products and preparations containing proteins. The majority of proteins contain several tyrosine residues, such as the pharmaceutical relevant proteins insulin, insulin growth factors, nerve growth factors, and tumor necrosis factors. If pharmaceutical preparations are exposed to ambient and artificial UV-light, long-term inter-molecular aggregation may occur via dityrosine cross-linking.

The irradiance level used in our experiments (2.20 W. m^−2^ at 276 nm) is similar to the total irradiance of sunlight in the UVB region (∼0.78 W. m^−2^, 280–315 nm) (See Table S1). UV sterilization lamps (Hg), which can be used during disinfection of the drug products, have irradiance levels ranging from 10 to 2400 W. m^−2^ at 254 nm, wavelength at which Tyr still absorbs (Table S1). Commercially available indoor light sources used commonly in industry, hospitals, warehouses, such as fluorescent tubes, quartz halogen lamps and even tungsten filament incandescent lamps can provide UVB (280–315 nm) and even UVC (200–280 nm) emission [62]. For instance, energy efficient compact fluorescent lamps (CFL) [63], [64] provide irradiance levels up to 2–3×10^−2 ^W.m^−2^ in UVB and 8.15×10^−3^ W.m^−2^ in UVC (see Table S1). Though the irradiation levels of CFL and other light sources are still lower than the used in our experiments and that the distance of measurement is still quite short (0.1–0.2 m), it would not be surprising that long exposure times of insulin preparation to the above cited light sources will also induce partial degradation of the protein.

The FDA (Food and Drug Administration) ICH Q1B (Photostability Testing of New Drug Substances and Products) guideline for industry does not include any recommendation for carrying out photostability tests for light sources below 320 nm, which excludes testing for Tyr absorption in drugs containing proteins [5]. Furthermore, in the “confirmatory study” approach present in the ICH Q1B guideline, an illumination level with an integrated near-UV energy of 200 Wh. m^−2^ (0.055 W. m^−2^) or more is recommended [5]. These irradiation levels might not represent accurately the exposure to UV-light suffered by drug products in industry.

It is important to include photostability studies of protein drug candidates prior to final formulation as also recommended by Rathore and Rajan [5]. The exposure time and spectral outputs of the light sources used should be investigated. During production, it is important to avoid the use of UV-light sources, including natural sunlight. The pharmaceutical preparations should be packed with UV-blocking materials.

## Supporting Information

Table S1 **Irradiance and other properties - experimental set-up, solar light and commonly used commercial light sources.**
(DOCX)
