# Supplementary material for: UV-Light Exposure of Insulin: Pharmaceutical Implications upon Covalent Insulin Dityrosine Dimerization and Disulphide Bond Photolysis
Source: PLoS One. 2012 Dec 5;7(12):e50733. doi: 10.1371/journal.pone.0050733 (PMC3515625; doi:10.1371/journal.pone.0050733)
Supplement: Table S1 — Irradiance and other properties - experimental set-up, solar light and commonly used commercial light sources. (DOCX) [file pone.0050733.s001.docx]

| **Source** | **Light source / Lamp type** | **Purpose** | **Wavelength (nm)** | **Irradiance (W.m^-2^)** | **Measurement Position** |
| --- | --- | --- | --- | --- | --- |
| Our experiments | Xenon Arc Lamp | - | 276^*^ | 2.20 | At cuvette position |
|  |  |  | 280^*^ | 2.31 |  |
|  |  |  | 285^*^ | 2.39 |  |
| Reference solar spectrum – Direct + Circunsolar Solar Spectral Irradiance  ASTM G173-03 Air Mass 1.5 Reference Spectra, American Society for Testing and Materials (ASTM) [65] | Sun | - | 282.5^**^ | 1.16x10^-16^ | Surface of earth |
|  |  |  | 285^**^ | 4.6x10^-13^ |  |
|  |  |  | 280-315 | 0.78 |  |
| Sen Lights Corporation [66] | Low and High Pressure Hg Lamps | UV sterilization | 254^***^ | 10 to 2400 | 1 meter distance from lamp |
|  | Cold Cathode Lamps | Illumination, neon signs | 254^***^ | 11 to 240 |  |
| Study on Compact Fluorescent Lamps from Khazova and O’Hagan [63]  73 commercial Compact Fluorescent Lamps were acquired from UK major retailers and tested | Compact Fluorescent Lamps  Single Envelope (1 lamp) |  | 254^**^ | ~2.6x10^-3^ | 0.2 m distance from lamp |
|  |  | Commercial, household, indoor lighting | 289^**^ | ~3.6x10^-4^ |  |
|  | Compact Fluorescent Lamps  Single Envelope (53 lamps tested) |  | 313^***^ | >0.01 (24 % of lamps tested) | 0.2 m distance from lamp |
|  |  |  |  | 0.005-0.01 (30%) |  |
|  |  |  |  | 0.001-0.005 (21%) |  |
|  |  |  |  | 0.0001-0.005 (21%) |  |
|  |  |  |  | <0.0001 (4%) |  |
|  | Compact Fluorescent Lamps  Double Envelope (20 lamps tested) |  |  | 0.001-0.005 (20% of lamps tested) | 0.2 m distance from lamp |
|  |  |  | 313^***^ | 0.0001-0.005 (40%) |  |
|  |  |  |  | <0.0001 (40%) |  |
| Study on Compact Fluorescent Lamps from Sharma et al. [64]  18 commercial Compact Fluorescent Lamps were tested | Compact Fluorescent Lamps  Single Envelope |  | 280-315 | 2.87x10^-3^ to 28.15x10^-3^ | 0.1 m distance from lamp |
|  |  |  | 200-280 | 1.39x10^-3^ to 8.15x10^-3^ |  |
|  | Compact Fluorescent Lamps  Double Envelope |  | 280-315 | 0.55x10^-3^ to 1.57x10^-3^ |  |
|  |  |  | 200-280 | 0.21x10^-3^ to 0.53x10^-3^ |  |
| Study on Compact Fluorescent Lamps from Nuzum-Keim and Sontheimer [67]  12 commercial Lamps were tested | Incandescent lamps (3 out of 5 lamps tested) |  | 280-315 | 2x10^-11^ to 3x10^-11^ | 0.1 m distance from lamp |
|  | Halogen lamp (1 out of 2 lamps tested) |  |  | ~2.5x10^-11^ |  |
|  | Unshielded Compact Fluorescent Lamps  (5 lamps tested) |  |  | 1x10^-11^ to 4.5x10^-11^ |  |
|  | Shielded Compact Fluorescent Lamps  (1 out of 2 lamps tested) |  |  | ~1x10^-11^ |  |

^1^The irradiance value was measured/calculated from the power measured in the instrument at each wavelength and considering the exposition area of the cuvette. The bandwidth in the excitation slit was 5 nm.

^2^The irradiance value at each specific wavelength (e.g. 276 nm) was calculated by integration the spectral irradiation values over bandwidth of 5 nm.

^3^The calculation or measurement method was not stated in the source.
